# The Influence of Palatable Diets in Reward System Activation: A Mini Review

**DOI:** 10.1155/2016/7238679

**Published:** 2016-03-20

**Authors:** Isabel Cristina de Macedo, Joice Soares de Freitas, Iraci Lucena da Silva Torres

**Affiliations:** ^1^Pharmacology of Pain and Neuromodulation Laboratory: Animal Models, Department of Pharmacology, Universidade Federal do Rio Grande do Sul, Institute of Basic Health Sciences, 90050-170 Porto Alegre, RS, Brazil; ^2^Graduate Program in Biological Sciences-Physiology, Universidade Federal do Rio Grande do Sul, Institute of Basic Health Sciences, 90050-170 Porto Alegre, RS, Brazil; ^3^Graduate Program of Pharmacology and Toxicology, Pontifícia Universidade Católica do Rio Grande do Sul, Institute of Toxicology, 90619-900 Porto Alegre, RS, Brazil

## Abstract

The changes in eating patterns that have occurred in recent decades are an important cause of obesity. Food intake and energy expenditure are controlled by a complex neural system involving the hypothalamic centers and peripheral satiety system (gastrointestinal and pancreatic hormones). Highly palatable and caloric food disrupts appetite regulation; however, palatable foods induce pleasure and reward. The cafeteria diet is such a palatable diet and has been shown consistently to increase body weight and induce hyperplasia in animal obesity models. Moreover, palatable high-fat foods (such as those of the cafeteria diet) can induce addiction-like deficits in brain reward function and are considered to be an important source of motivation that might drive overeating and contribute to the development of obesity. The mechanism of neural adaptation triggered by palatable foods is similar to those that have been reported for nondrug addictions and long-term drug use. Thus, this review attempts to describe the potential mechanisms that might lead to highly palatable diets, such as the cafeteria diet, triggering addiction, or compulsion through the reward system.

## 1. Introduction

Currently, an important cause of obesity has been observed to be related to changes in eating patterns that have occurred in recent decades [1]. The daily consumption associated with so-called Western diets consists of highly palatable and caloric food [2], and such diets have become a habit that has led many individuals to develop obesity [3]. Recent studies using the cafeteria diet as an experimental model of obesity with or without associated chronic stress have shown that animals exposed to this diet became obese and exhibit important changes in lipid profiles, endocrine appetite markers, and the development of hyperphagia [4, 5].

Food intake and energy expenditure are thought to be controlled by complex neural systems, and the hypothalamus has been recognized as the center of homeostatic regulation (for review see [6]); however, palatable foods, such as those of the cafeteria diet, can lead to impairments of normal appetite regulation [7]. In addition, palatable food disrupts appetite regulation and induces pleasure and reward. Excessive consumption of palatable energy-dense food can lead to a profound state of reward hyposensitivity that is similar to that of drug abuse that can lead to the development of compulsive-like eating [8].

Based on recent evidence that suggests that nondrug addictions might lead to neural adaptations similar to those that have been reported with long-term drug use, this review attempts to describe the putative mechanisms that might lead to the triggering of addiction or compulsion by highly palatable diets, such as the cafeteria diet, through the reward system.

## 2. Food Control Integration

Food control is a complex mechanism that involves the appetite, motivation, and energy demands of the organism and these aspects can be modified by food availability and exposure. The central nervous system detects a wide variety of peripheral neural and humoral markers, and this complex neural network receives endocrine and hormonal inputs. Hormones, such as leptin, insulin, pancreatic polypeptide (PP), amylin, ghrelin, cholecystokinin, glucagon-like peptide (GLP-1), and oxyntomodulin, coordinate food intake through signaling and modulation in orexigenic and anorexigenic neurons (for review see [9]). These markers reflect gastrointestinal functions and energy needs, including taste, which is a central factor in decision-making related to feeding behavior, and the olfaction. Both functions are capable of discriminating features such as odor, texture, and temperature and participating in the choice of food to be ingested [10]. The homeostasis regulation and maintenance of stable body weight depend on the integration of these signals and on the ability to respond appropriately through modulation of energy expenditure and food intake [11]. Hypothalamic centers control food intake and weight gain and are part of a complex of neuroregulatory interactions that include the peripheral satiety system (gastrointestinal and pancreatic hormones) and a large-scale central neural network [12]. The importance of the hypothalamus in energy homeostasis was first suggested by classic lesioning experiments performed rodents, and subsequent studies suggested the roles of hypothalamic nuclei, such as the arcuate nucleus (ARC), paraventricular nucleus (PVN), ventromedial nucleus (VMN), dorsomedial region (DMV), and lateral hypothalamic area (LHA), in energy homeostasis [13]. The blood-brain barrier (BBB) adjacent to the ARC region serves as the interface of the peripheral metabolic signals and the brain. While the DMV area is the region of satiety, the LH nuclei are the main controllers of feeding responses [14]. Damage to the hypothalamus, particularly the lateral and dorsomedial hypothalamus, disrupts feeding behavior [15]. Food intake and energy metabolism are regulated by a complex interaction between orexigenic and anorexigenic neuropeptides in the ARC of the hypothalamus and peripheral tissues. Neuropeptide Y (NPY) and agouti-related protein (AgRP) are coexpressed in neurons of the ARC and are potent orexigenic peptides. Additionally, the *α*-melanocyte-stimulating hormone (*α*-MSH) and cocaine- and amphetamine-regulated transcript (CART) peptide are potent anorexigens [16]. The hypothalamic nucleus receives inputs of several peripheral hormones including leptin; for example, the arcuate nucleus of the hypothalamus and the area postrema of the nucleus tractus solitarius express leptin receptors and are important regions of appetite control and food ingestion. Leptin is a hormone that is synthesized and released by adipose tissue and acts as food control in the ARC of the hypothalamus. This hormone stimulates neurons to secrete proopiomelanocortin (POMC), which is a precursor protein of *α*-MSH that also stimulates POMC neurons to secrete CART. Leptin also inhibits AgRP/NPY neurons, which coexpress the orexigenic neuropeptides AgRP and NPY, and antagonizes *α*-MSH. The combined effect of the actions of leptin suppresses appetite and contributes to the maintenance of energy homeostasis (for review see [17]). Another important hormone that is related to food control is ghrelin. This hormone is produced by the stomach, hypothalamus (ARC and infundibular nucleus), and pituitary gland. After being released into the blood stream, ghrelin reaches the ARC and activates NPY and AgRP neurons, which leads to increased food intake [18]. In addition to acting on dietary control, both leptin and ghrelin are involved in the reward system [17, 18]. Leptin receptors are also found in the mesolimbic pathway in the reward-associated ventral tegmental area (VTA) and the substantia nigra [19]. Thus, leptin influences the hedonic aspects of feeding and interacts with the mesolimbic-dopaminergic system, which is known to regulate arousal, mood, and reward (for review see [17]), while ghrelin stimulates dopamine neurons in the ventral tegmental area (VTA) and promotes dopamine turnover in the nucleus accumbens of the ventral striatum, which is part of the major central reward pathway (for review see [18]). Accordingly the balance between food control centers and peripheral signals determines appetite and energy expenditure and influences the reward system.

## 3. Palatable Foods and the Reward System

Palatable foods with high fat and sugar contents are associated with increased food intake [7, 20]. Palatable foods alter the behavior of experimental animals. In a study of obese rats with histories of extended access to palatable food, the rats were found to continue to eat palatable food even in the presence of a noxious light cue that predicted the delivery of an aversive foot shock [7]. Moreover, mice that have previously had access to a palatable high-fat diet spend more time in an aversive environment to obtain the palatable food than do mice with no prior experience of the diet [21].

Highly palatable foods activate the reward system to affect feeding behavior [22]. From evolutionary perspective, these foods that are high in fat and sugar are more attractive because they can be quickly converted into energy [23]. The consumption of these foods over a long period of time can be compared to drug addiction [24] mainly because these foods generate progressive increases in food intake [25] that lead to a phenomenon that is comparable to the adaptation triggered by drugs [26]. In addition, the macronutrients of the palatable food can stimulate the brain reward systems independently of their caloric value [27]. High levels of consummatory behavior are induced by drugs abuse such as cocaine or nicotine despite the fact that these drugs are devoid of caloric or nutrient value [28]. Extended access to palatable high-fat food, such as the cafeteria diet, can induce addiction-like deficits in brain reward function that are thought to be important sources of the motivation that might drive overeating and contribute to the development of obesity [8].

The cafeteria diet is one of many animal obesity models and involves a palatable diet that uses the human foods, such as biscuits, wafers, condensed milk, sausages, and soft drinks. These foods have high sugar, salt, and spice, contents that make them highly palatable, and palatability is critical for determining food preference [29]. Moreover, this diet has been shown to consistently increase body weight, induce hyperphagia, and alter the metabolic factors related to the metabolic syndrome cluster [2, 4–6, 20, 30, 31]. Indeed, this diet is one of the factors that has contributed to rapid increase in obesity over the past thirty years [32]. The cafeteria diet mimics modern patterns of human food consumption and was adapted from a diet that is also known as the Western diet and was previously described by Estadella et al. (2004) [20]. Preference for the cafeteria diet over standard chow has been shown in studies with obesity models [2, 32, 33]. Furthermore, the cafeteria diet, along with other palatable diets, acts on many neurotransmitter systems and can lead to changes in the reward system [2].

Brain regions, such as the lateral hypothalamus (LH), nucleus accumbens (NAc), ventral tegmental area (VTA), prefrontal cortex (PFC), and amygdala, are activated in response to palatable food. There is also a connection between the nucleus accumbens (NAc) and the lateral hypothalamus (LH) that is important for energy homeostasis (for review see [7]). The LH is also functionally connected to other cortical and limbic brain sites that have been implicated in organizing and directing behavior toward obtaining palatable food. LH damage abolishes the stimulatory effects of NAc manipulations on food intake, while inactivation of the NAc enhances the activity of the LH, particularly LH neurons [34]. The NAc is a brain region that seems to play a crucial role in behavior related to feeding and drug reward [35]. This structure is considered to serve as an interface of emotion, motivation, and action based on its numerous inputs from the amygdala, prefrontal cortex (PFC), and hippocampus (for review see [36]). The NAc receives information from the brain stem in response to ingested food through a connection with the nucleus of the solitary tract (for review see [36]). The NAc receives information from the brain stem in response to ingested food through a connection with the nucleus of the solitary tract (for review see [37]). It is important to note that nucleus accumbens has been subdivided into medioventral shell (NAcs) and a laterodorsal core (NAcc) in accordance with morphological features, and its different projections were studied with tract-tracing methods. Thereby depending on the specific places of the nucleus accumbens where dopamine transmission is released, different behavioral responses can be triggered [38, 39]. In addition, the amygdala is a key structure for the processing of emotions and integrates food-related sensory and physiological signals from the hindbrain and cortex (for review see [36]). The amygdala connects external and internal sensory information with the motivational systems of the brain and sends input to the NAc. The hippocampus has crucial roles in memory formation and in the control of food intake, while the prefrontal cortex (PFC) is responsible for higher-order cognitive processing, planning, and decision-making. The PFC receives input from insular cortical regions that relay gustatory information and has an important influence on NAc signaling. The neurons that connect the brain regions involved in reward behavior are related to many neurotransmitter systems. Moreover, studies have shown that dopamine, endogenous opioids, and serotonin are highly related to drug and food addiction (for review see [7]).

## 4. Neurotransmitters Involved in the Reward System

### 4.1. Dopamine

Dopamine (DA) is a neurotransmitter that has been more extensively implicated in the mechanism of drug addiction due to its influence on neuroadaptation and psychostimulant reward process [40]. Studies employing microdialysis technique showed that addictive substances increase extracellular dopamine (DA) release in the NAcc [37] and the changes in dopamine transmission in the NAcs and NAcc in response to appetitive and consummatory behavior motivated by food [38]. Dopaminergic neurons are located in the midbrain; they send their axons through the medial forebrain bundle and innervate wide regions within the systems while dopaminergic reception and the intracellular signaling are mediated through the two major subtypes of G protein-coupled DA receptors [41]. It is important to consider that dopamine receptors regulate signaling cascades on cells that can alter the transcription of genes and can trigger neuroadaptative and behavioral changes on brain structures with changes in protein synthesis. This way, the learning theories of addiction postulate that some psychostimulant substances are engaged on molecular mechanisms implicated in learning and memory as D1 receptors and downstream intracellular messenger cascades that may cause synaptic rearrangements. Likewise, these substances induced dopamine release and may alter learning-related molecular changes by activating common signal transduction pathways. Several studies showed that psychostimulant substances are related to memory consolidation, and it suggests that addiction is due to drug-induced neuroadaptations in reward-related learning and memory processes in the NAcc [42].

The corticolimbic pathways that are responsible for reward-associated feeding behavior include the ventral tegmental area, insular cortex, anterior cingulate cortex, orbitofrontal cortex [13], substantia nigra, amygdala, prefrontal cortex, posterolateral ventral striatum (globus pallidus and putamen), and anteromedial ventral striatum (nucleus accumbens and caudate nucleus) [17]. Within the NAc, GABAergic medium spiny projection neurons (MSNs) are divided into those that express the dopamine 1 receptor (D1R) and project directly back to the VTA (direct pathway) and those that express the dopamine 2 receptor (D2R) and project back disynaptically after first impinging onto the ventral pallidum (VP). The excitation of striatal D1R-MSNs is associated with reinforcing behavior, whereas the activation of striatal D2R-MSNs exerts the opposite effect [43, 44]. The mesolimbic and the mesocortical pathways regulate the dopamine (DA) systems effects on reward-related behavior, and modifications of these systems are associated with the rewarding effects of drugs and food [45].

Drug abuse and palatable food with high fat and sugar content can significantly activate the DA reward circuitry, and both increase dopamine levels in the mesolimbic system and dopaminergic transmission in the NAc [45]. For example, microdialysis studies in the rat showed that appetitive taste stimuli release DA in the NAcs, NAcc, and prefrontal cortex (PFC). However, DA responsiveness is different among these structures and it depends on hedonic, taste, and novelty stimulus. In addition, single exposure to palatable food in NAcs promptly induces habituation of DA responsiveness, consistent with a role in associative learning. However, this effect does not occur in NAcc and PFC. It is important to note that mild food deprivation can impair habituation of NAcs DA responsiveness to palatable food. It has been suggested that DA release in this region is not the cause but consequence of the food reward. The taste properties of food can have good or bad postingestive consequences which are related to DA release of NAcs after food intake [46].

**Figure 1:**
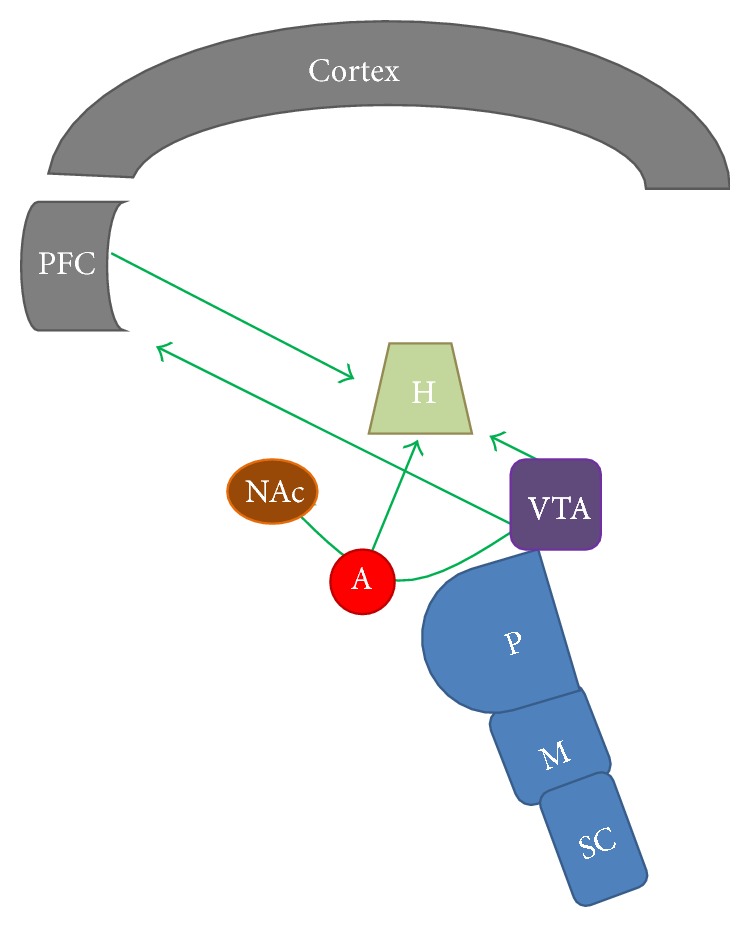
The dopaminergic pathways involved in food control. Dopamine neurons in the VTA send axonal projections to H, A, NAc, and PFC. The projections of the dopaminergic system from A and PFC to LH are directly involved in the regulation of food intake regulation. SC: spinal cord; M: medulla oblongata; VTA: ventral tegmental area; PFC: prefrontal cortex; A: amygdala; NAc: nucleus accumbens; H: hypothalamus.

It should be noted that dopamine is associated with reward related to food intake and the behaviors required to maintain feeding for survival. Dopamine-deficient (DA−/−) animals with inactivations of the tyrosine hydroxylase gene in dopaminergic neurons develop fatal hypophagia; however, if dopamine is replaced in the caudate/putamen or the NAc of such animals, they commence feeding but only show interest in sweet foods and palatable chow [47]. Additionally, ghrelin, orexins, and NPY can act as modulators of the mesolimbic DA system. These peptides might change the frequencies or patterns of the action potentials generated in the dopaminergic cells of the VTA or induce downstream DA release in the NAc [14]. Chronic drug abuse induces dopaminergic stimulation that results in impaired inhibitory control, compulsive drug intake, and enhanced emotional reactivity to drugs. Similarly, repeated exposure to high fat and sugar content foods results in compulsive food consumption, poor control of food intake, and food stimulus conditioning [48]. Midbrain dopamine transmission influences palatable food intake in humans. For example, Parkinson's disease (PD) induces degeneration of dopamine-containing neurons in the midbrain, and patients treated with dopamine receptor agonists can exhibit compulsive-like palatable food consumption; even non-PD affected human subjects can exhibit hedonic over eating following the administration of DA receptor agonists. The dopamine pathway is activated in humans and laboratory animals in response to palatable food and appetitive food-related cues. In addition, leptin, ghrelin, and other regulators of appetite influence the activity of system, which suggests that the midbrain dopamine systems play an important role in palatable food consumption (for review see [34]). Indeed, dopaminergic pathways are heavily involved in the reward system. Dopamine neurons in the VTA send axonal projections to the amygdala, nucleus accumbens, and prefrontal cortex. The projections of the dopaminergic system from the amygdala and prefrontal cortex to the lateral hypothalamus, as shown in Figure 1, are directly involved in food control [34].

### 4.2. Opioid System

The endogenous opioid system is also related to reward, addiction, and eating behaviors, and the roles of endogenous opioid peptides, such as *β*-endorphin and enkephalins, in producing reward, are well established [49]. The endocannabinoid and opioid systems have wide receptor distributions within the CNS and play important roles in reward-related feeding [50, 51]. In mammals, the endogenous opioids derived from POMC, which is a precursor of opioids including *β*-endorphins, that bind to opioid receptors that are distributed in the hypothalamic regions are involved in the control of food intake (for review see [7]). Morphine has a strong rewarding effect and addiction liability. Morphine's rewarding action is mediated via the mesolimbic-dopaminergic pathway extending from the VTA to the NAc [52]. Studies have shown that infusions of *μ*-opioid receptor agonists, such as DAMGO, into the NAc stimulate feeding behavior in rats with ad libitum access to food [53], and opioid receptor antagonists infused into the NAc decrease the consumption of preferred food without affecting the intake of less palatable alternatives (for review see [34]). In addition, systemic injection of a *μ*-opioid antagonist prevents the stimulatory effect of palatable food on dopamine release in the NAc [54]. Moreover, morphine enhances the frequency of the firing of mesolimbic dopamine neurons in the VTA and increases dopamine turnover in the NAc, which confirms the excitatory effects of opioids on the dopamine system [55–57]. Regarding the cannabinoids, evidence suggests that the cannabinoid-1 (CB1) receptor has a role in the rewarding aspects of eating. The peripheral administration of CB1 antagonists reduces the intake of palatable sugar in rats [58, 59]. Cannabinoid receptor (CB1) antagonist administration prevents the orexigenic effect of the endocannabinoid agonist anandamide on food intake [60]. Leptin reduces endocannabinoid levels in the hypothalamus, which suggests that hypothalamic endocannabinoids might act via CB1 to increase food intake through a leptin-regulated mechanism [13].

### 4.3. Serotonin

**Figure 2:**
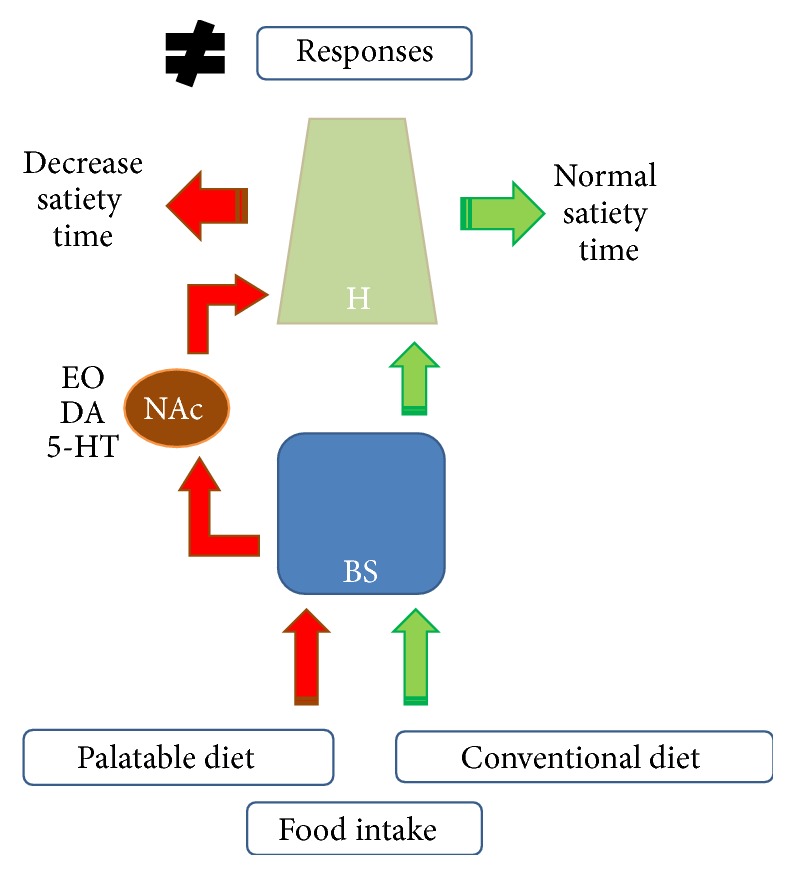
Signaling of food intake in the brain. The signaling pathway activated by a conventional diet is shown on the right (green), whereas the signaling induced by a palatable diet is shown on the left (red). H: hypothalamus; NAc: nucleus accumbens; BS: brain stem. EO: endogenous opioids; DA: dopamine; 5-HT: serotonin.

Serotonin or 5-hydroxytryptamine (5-HT) is known as a modulator of feeding behavior and satiety signals. In the hypothalamus, this neurotransmitter inhibits the expression of NPY to reduce hunger [7, 61, 62]. This mechanism might be the link between 5-HT and appetite regulation. Drugs that either induce the release of 5-HT (e.g., d-fenfluramine) or inhibit its reuptake (e.g., fluoxetine, sertraline, and sibutramine) and agonists of the 5-HT1B and/or 5-HT2C receptors inhibit food intake [63, 64]. The consumption of palatable foods, which have more intense flavors than standard foods, sends information to the reward center in the nucleus accumbens, which triggers dopamine and serotonin release. The reward center has connections with neurons in the hypothalamus that act on appetite control. Thus, highly palatable diets increase the time required to reach satiety, which leads to an increase in food consumption, which in turn can lead to overweight and obesity [7]. There are enhanced demands for serotoninergic and dopaminergic signaling in the reward systems of overweight subjects, and these features might lead to increased motivation for food consumption. The implication of reward centers in eating behavior supports the hypothesis that obesity and drug addiction share common mechanisms [65]. Appetite regulation, food intake, and diet are closely interconnected with mood regulation, and obesity has been identified as environmental risk factor for affective psychiatric disorders, including anxiety and depression. Moreover, major depression in adolescence is linked to a greater risk for obesity in adulthood, and these metabolic conditions might be exacerbated in depression. Similarly stress exposure significantly affects food intake in humans and animals and might promote metabolic disturbances, hyperphagia, and consequent obesity. Moreover, acute stress responses are reduced following the intake of palatable rewarding foods, which potentially explains the phenomenon of “comfort eating” that has been observed in individuals as self-medication for stress relief (see [66] for review). In summary, the NAC (reward center) receives inputs of endogenous opioids, serotonin, and dopamine and sends outputs to neurons of the hypothalamus that act on appetite control. Unlike conventional standard diets, highly palatable diets are slower to induce satiety [67], which results in increased food intake that can lead to overweight and obesity as shown in Figure 2.

## 5. Conclusions

Obesity is a global pandemic and major health burden with the associated risk factors of cardiovascular disease and diabetes mellitus. The current dietary patterns predominantly include high calorie foods that are high in fat and sugar as exemplified by the cafeteria diet, which has been used as an animal model. Such diets unleash pleasure and lead to drastic increases in food intake. These foods lead to disruptions of several signaling pathways that are related to food control, including activation of the reward system. Thus, palatable foods lead to addiction through mechanisms that are similar to those of drugs of abuse. This scenario increases the level of difficulty related to the planning and development of new pharmacological strategies for obese patients.
